# Evolution of joint cooperation under phenotypic variations

**DOI:** 10.1038/s41598-018-22477-5

**Published:** 2018-03-07

**Authors:** Te Wu, Long Wang, Joseph Lee

**Affiliations:** 1 Center for Complex Systems, Xidian University, Xi’an, China; 2 Department of Applied Mathematics, The Hong Kong Polytechnic University, Hung Hom, Hong Kong China; 3 Center for Systems and Control, College of Engineering, Peking University, Beijing, China

## Abstract

Effects of phenotypic variation on the species-environment systems and the evolution of cooperation under prescribed phenotypic diversity have been well addressed respectively. Interspecies interactions in the context of evolvable phenotypic diversity remain largely unconsidered. We address the evolutionary dynamics by considering evolvable phenotypic variations under group interactions. Each individual carries a capacitor of phenotypes and pays a cost proportional to its volume. A random phenotype from the capacitor is expressed and the population is thus divided into subpopulations. Group interactions happen in each of these subpopulations, respectively. Competition is global. Results show that phenotypic diversity coevolves with cooperation under a wide range of conditions and that tradeoff between expanding capacitor and rising cost leads to an optimal level of phenotypic diversity best promoting cooperation. We also find that evolved high levels of phenotypic diversity can occasionally collapse due to the invasion of defector mutants, suggesting that cooperation and phenotypic diversity can mutually reinforce each other.

## Introduction

To understand the emergence and persistence of cooperative behavior among selfish individuals, a variety of mechanisms have been proposed. One intensively studied is the tag based cooperation. Riolo *et al*. have originally constructed a model with continuous tags^1^, but does not count the full defection as a possible strategy^2^. In ref.^3^, Traulsen and Schuster have simplified and presented the basic aspect of, the original model^1^. Later they extended this simplified model^3^ to the spatial settings^4^. Jansen and van Baalen^5^ have explored the chromodynamics on lattice-structured populations. One gene encodes for tag and another for strategic behavior. It has been shown that tight coupling of strategy and tag inhibits tag diversity, leading to the low level of altruism. Loosening the coupling to some degree but not too much induces the co-existence of many tags and thus upgrading altruism to high levels. In another work, Traulsen and Nowak have considered the chromodynamics in well-mixed populations of finite size^6^.

These studies have involved two key aspects, tags (or phenotypes) and contingent cooperation^1–10^. Phenotypes serving as signals are observable and thus individuals are able to base their strategic behaviors on observed phenotypes of opponents. In these models^1–10^, a set of phenotypes are available. Once the set is pre-assigned, it does not evolve over time and thus the phenotypic diversity is fixed. Individuals have an equal access to each of these phenotypes. Natural questions arise which levels of phenotypic diversity would be selected if the phenotypic diversity itself evolves, and if cooperation can get stabilized under these selected levels?

On the other hand, recent experimental studies^11–14^ have shown that genetically identical cells are capable of exhibiting different phenotypes, and a single cell can switch between distinct phenotypes^15–19^. By phenotype switching, the population can either survive fluctuating environments^16,18^, or maximize fitness^19^, or preserve some properties^15,17^. These observations have at least three implications: individuals carry redundant phenotypes; individuals have the ability to switch phenotypes; some phenotypes are more fit than others in some environment while it turns around when environment changes. Moreover, these studies^11,12,15–19^ have well captured the species-environment interactions but seldom dealt with direct inter-species interactions.

In fact, when cooperators cooperate probabilistically, the cooperative act itself seen as phenotype exhibits variations^20,21^. In ref.^20^ phenotypic variations differentiate the cooperation gene carriers into those who have expressed (i.e., having cooperated) the gene and those who have not. The later can benefit from their genetically identical species’s contributions as defectors do. When the population is structured by an infinite number of demes, this phenotype-mediated interactions suffice to evolve cooperation. This claim is borne out experimentally by regarding TTSS-1 expression as phenotypic trait. Another experimental research^21^ has also confirmed that cooperative virulence can be stabilized by the expression of an avirulent phenotype. Even cheaters cheat facultatively in the social amoeba *Dictyostelium discoideum*^22^. We would like to emphasize that in these studies phenotype describes strategic behavior but does not serve as signal. Interestingly, the authors^23^ have pointed out that under greenbeard mechanism, an allele can play dual roles of recognition and thus preferentially directing the benefits to copies of itself in others.

**Figure 1:**
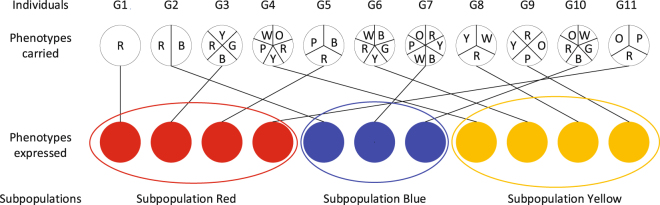
Phenotypic diversity and local interactions. As phenotypic variation is considered individuals may vary in capacity of carrying phenotypes. Each individual expresses one phenotype at random from those he has carried. Only phenotypes expressed are visible. According to the phenotype expressed, the population is divided into a certain number of subpopulations, each consisting of individuals expressing the same phenotype. In this schematic illustration, there are 11 individuals, say *G*1, *G*2, …, *G*10 and *G*11. They are located in three subpopulations based on phenotypes expressed: Subpopulations Red, Blue and Yellow. For instance, individual *G*1 just carries one phenotype Red and expresses this phenotype. Individual *G*11 carries three potentially expressible phenotypes, say Red, Blue,and Orange and happens to express Red. So *G*1 and *G*11 belongs to the same subpolulation Red and have chance to play the public goods games. Following the same logic, one can grasp other individuals in term of phenotype carrying and expressing. Obviously, individuals carrying more phenotypes are more likely to enter each of these subpopulations. But as each individual needs to pay a cost proportional to the number of phenotypes carried, those individuals who carry too many phenotypes would be selected against. When the size of the subpopulation is less than the group size as required for the public goods game to happen, no interaction takes place in this subpopulation. At this time, fitness is uniquely determined by the cost of retaining capacitor and expressing phenotype.

We shall construct a model incorporating the evolvable phenotypic variations and strategic behaviors to address the resulting coevolutionary dynamics. We shall consider a well-mixed population of finite size (=*N*). Each individual is well defined by three properties: a capacitor of potentially expressible phenotypes, phenotype expressed, and strategic behavior. Capacitor prescribes which phenotypes can be expressed. Each individual randomly expresses one phenotype from her capacitor. This phenotype expressed serves as signal and is observable. Strategic behavior defines whether or not to cooperate. Each individual needs to bear a cost to retain capacitor and express phenotype. According to the phenotype expressed, the population is divided into a number of subpopulations, each consisting of individuals expressing the same phenotype. Cooperators therefore can tunnel their help towards those of the same phenotype. This is perfectly captured by limiting public goods interactions to each subpopulations (For more details see Model description and Fig. 1). Unlike previous models, our model does not preassign the level of phenotypic diversity. Rather we let it be an evolvable trait. This setting allows us to probe and answer questions previously asked. In the limit of small mutation rates, the population dynamics can be approximated by an embedded Markov chain. Our results show that cooperation and phenotypic diversity can coevolve within a wide range of conditions, and moreover, natural selection favors an optimum level of phenotypic diversity.

## Results

**Figure 2:**
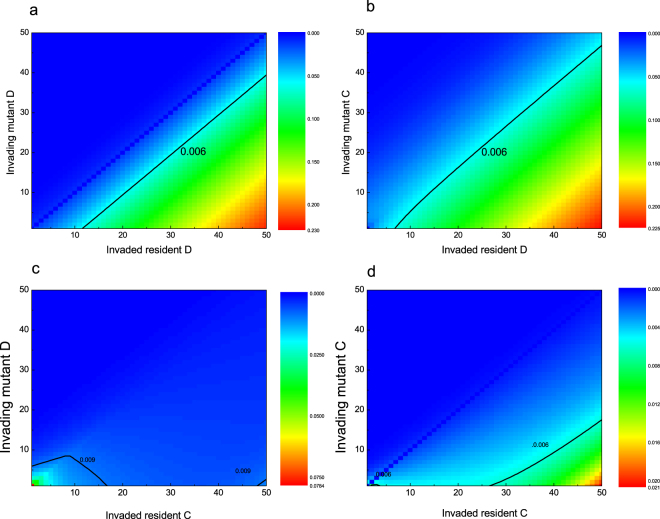
The pairwise invasion dynamics. Transition rate means the probability that the population moves from an invaded state to an invading state. In this evolutionary process, no new mutation occurs. Just two subpopulations, one consisting of the mutants, the other the residents, compete to survive. The capital letter, *C* or *D*, along the *Y*-axis, denotes the mutant’s behavioral strategy, while the one along the *X*-axis denotes the residents’ behavioral strategy. The coordinate value denotes the volume of capacitor. Parameters: *N* = 40, *r* = 3.2, *g* = 5, *β* = 0.1, *θ* = 0.12.

Let us start with the pairwise invasion dynamics, which shall be profitable for understanding the full population dynamics. When the mutant defectors attempt to invade the resident defectors, they incur no cost and generate no public goods in terms of the game interaction, but still burden the cost of retaining capacitor and expressing phenotypes. Larger volume of capacitor leads to a higher cost. Thus those defectors who carry more potentially expressible phenotypes put themselves in a more unfavorable position. This explains that the plot peaks at the bottom right corner and cascades downward towards the top left corner (Fig. 2a).

In fact, this property can be rigorously verified. Suppose that the invading defectors carry capacitor of volume *K*_*Y*_ and the invaded defectors carry capacitor of volume *K*_*X*_. Irrespective of the population composition and their actual phenotype expressions, the fitness is ${e}^{-\beta \theta {K}_{Y}}$ and ${e}^{-\beta \theta {K}_{X}}$, for an invader and a resident, respectively. It can be easily obtained that the fixation probabilities read as ${\rho }_{Y\to X}^{s}={\rho }_{Y\to X}^{d}=\frac{1-{e}^{\beta \theta ({K}_{Y}-{K}_{X})}}{1-{e}^{N\beta \theta ({K}_{Y}-{K}_{X})}}$. Using Equation (1) as presented in Methods Section, we can get the transition rate from state *X* to state *Y* as $r(X,Y;{K}_{X},{K}_{Y})=\frac{1}{2}{\rho }_{Y\to X}^{d}=\frac{1}{2}\cdot \frac{1-{e}^{\beta \theta ({K}_{Y}-{K}_{X})}}{1-{e}^{N\beta \theta ({K}_{Y}-{K}_{X})}}$ for both *K*_*X*_ > *K*_*Y*_ and *K*_*X*_ ≤ *K*_*Y*_. This transition rate decreases with *K*_*Y*_ and increases with *K*_*X*_, respectively.

Similar trends are observed when the mutant cooperators compete with the resident defectors. Obviously, before cooperators increase to the minimal number as required for the public goods game to happen, these cooperators fare like defectors. Due to neutral drift, once cooperators grow to the extent that they can play games, they can accrue payoffs. These payoffs compensate the cost of retaining the capacitor and expressing phenotypes and thus give them an upper hand in competing with defectors. As a result, cooperators share a higher chance when attempting to invade defectors than defectors do (Fig. 2b).

Interesting scenarios appear when the mutant defectors compete with the resident cooperators. As is well established, natural selection favors defection over cooperation in well-mixed populations. Inherent phenotypic diversity offers cooperators with potential opportunities to escape the chase and exploitation of defectors. To do this, cooperators need to expand their capacitors, which undoubtedly raises the cost of carrying increasing phenotypes. So how many phenotypes should cooperators carry? Fig. 2c shows that there exists an optimal interval in terms of capacitor’s volume. The interval is optimal in two senses that mutual breed between cooperators are able to counteract additional cost due to carrying more phenotypes, and that cooperators can effectively ward off direct competition with defectors. On the whole, cooperators possessing such capacitors enjoy the strongest resistance against defectors’ invasion, represented by the dependence of the transition rate on *K*_*C*_ in a *U*-shaped way. Meanwhile, the transition rate increases on either side of this interval. Reasons differ. At the left-hand side, cooperators carry fewer phenotypes, which can be quickly ‘deciphered’ and thus invaded. At the right-hand side, rising cost of expanding capacitor destroys the survival of these cooperators themselves.

The invasion dynamics shows similar sensitivity on *K*_*C*_ when the mutant cooperators attempt to invade the resident cooperators. Before the invading cooperators are likely to play the public goods games, they compete with the resident cooperators as if they were defectors. Once they expand to the group size, the evolutionary race goes another way. From then on, these invading cooperators can also benefit from reciprocity. Especially when the resident cooperators possess large-volume capacitors, the competitive advantage of the invaded cooperators over the invading cooperators is not significant or even non-existential, thus offering the invading cooperators much time of space to expand. Therefore at this time as invaders, cooperators can invade the resident cooperators more easily than defectors do (Fig. 2d).

**Figure 3:**
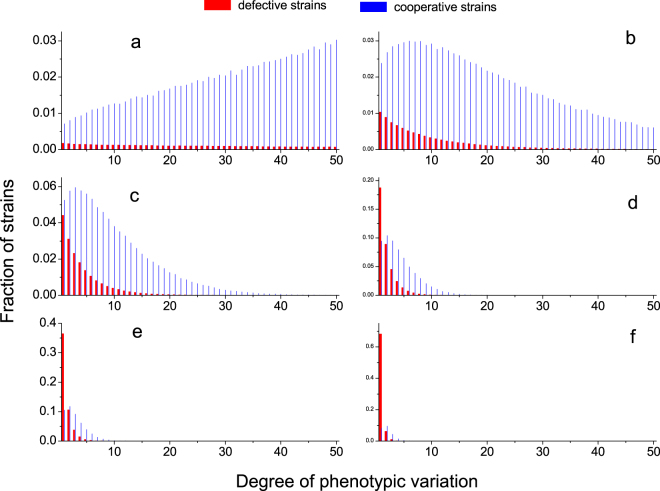
Stationary distribution for 2*M* competing strains. The bars are obtained by solving the eigenvector of the 2*M* × 2*M* transition matrix. Empty circles and empty triangles are obtained by simulations. Blue denotes cooperative strain, and red defective strain. The abscissa value represents the volume of capacitor. The evolutionary process is fully characterized in the main text. Parameters: *N* = 40, *r* = 3.2, *β* = 0.1. In **a,b,c,d,e,f**, *θ* is 0, 0.05, 0.12, 0.3, 0.5, 1, correspondingly, and the overall cooperation levels are 0.949, 0.909, 0.820, 0.623, 0.468, and 0.242, respectively.

With the elaborate elucidation of the pairwise invasion dynamics, we are now able to analyze the full population dynamics. Consider the competition of an arbitrary number of strains (=2*M*) in the well-mixed population of finite size. In the limit of rare mutations, the transition rates between any two strains have been analytically derived (see Equation (1) in Methods Section), defining an embedded Markov chain. By the chain we can compute the stationary distribution of the population, corresponding to the fraction of the time that the population spends in each of these 2*M* homogeneous states in the long run. We have corroborated by numerical simulations that our main results show no qualitative change for a wide range of mutation rate. In particular, the overall cooperation level is 0.5024, 0.6992, 0.8107 corresponding to the mutation rate 0.5, 0.05, and 0.01 respectively, as other parameters are kept the same as in Fig. 3c.

Results are presented in Fig. 3, where we plot the equilibrium level of these 2*M* strains as the parameter *θ* varies. Except for *θ* = 0, the distribution reveals general features. For defective strains, the larger capacitor they possess, the lower their fraction in the long. In other words, fractions monotonically decrease with expanding capacitor, a feature invariable with the change of *θ*. When it comes to cooperative strains, there exists an optimal solution in terms of phenotypic variations, associated with the capacitor’s volume *K*_*C*_ whose corresponding strain accounts for the highest fraction of all cooperative strains. Cooperative strains next to *K*_*C*_ also enjoy reletively high fractions. Moving away from *K*_*C*_ towards either side, the fraction of cooperative strain ramps down. This optimal volume lies in between one and *M*, the capacitor’s upper boundary. It is subject to *θ*. Growth in *θ* reduces the fraction of each type of cooperative strain and this optimal volume. Of interest, as *θ* expands to as high as 0.3 (Fig. 3d), defective strain with capacitor just covering one phenotype, especially outperforming the cooperative strain with capacitor covering *K*_*C*_ phenotypes, attains the highest fraction of all strains. Even so, the overall cooperation level can be still higher than the overall defection level. Therefore, the cooperation can be preserved and selected by diversifying inherent phenotypes.

Intuitions responsible for the emergence of an optimal level of phenotypic diversity are interesting and fundamental. As mutation is uniform and unbiased, it is the evolutionary force that uniquely determines the eventual fate of strains. Resident defective strains possessing capacitors of large volume can be easily invaded by defective strains, or by cooperative strains, when both just possess capacitor covering a small number of phenotypes in the evolutionary process. Occasionally it may happen that defective strain attempts to upgrade its capacity in expressing phenotypes, but as soon as it does so it will be pulled back. As a consequence, defective strains most of the time are stuck in low levels with respect to phenotypic variations. This undoubtedly limits the ability of defective strains in chasing and exploiting cooperative strains.

This, however, does not mean that the larger capacitors cooperative strains possess, the more they will be favored by selection. In fact, when carrying large numbers of phenotypes is prohibitively expensive, though cooperative strains are able to escape stalking of defective strains, mutual breed between themselves does not suffice to offset the cost of carrying so many phenotypes. These cooperative strains will be in a disadvantage place. When cooperative strains possess capacitors covering a small number of phenotypes, cost of carrying phenotypes does decrease. But the phenotypes they can express are so few that they can be readily ‘deciphered’ by defective strains who also possess volume-similar capacitors, and thus quickly be invaded. In between comes the optimal phenotypic variations for cooperative strains to carry. When cooperative strains’ capacitors with volume around *K*_*C*_ they can effectively dodge defective strains’ chasing and exploitation. Meanwhile, the cost of carrying such phenotypes can still be eclipsed by their mutual breed. So once these cooperative strains dominate the population, the evolutionary dynamics are quite stable in the sense that the dominance persists longer than other strains do.

**Figure 4:**
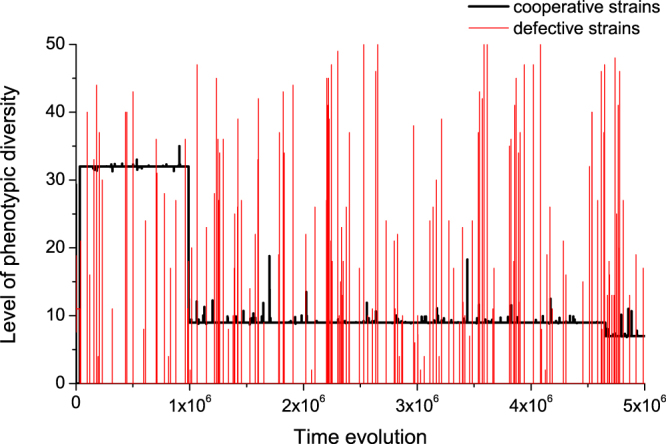
Time evolution of the competition between cooperative strains and defective strains. When the population is occupied by defective strain, it is most likely that the defective strain possesses capacitors of small volume. It is either followed by the invasion of defective strain with capacitors of similar volume, or by cooperative strain possessing capacitors of moderate volume. In the former case, the evolutionary process advances just as it starts. In the later case, it gets very hard for the cooperative strain to be invaded, since it possesses the strongest resistance power against invasion of other strains. In the average sense, cooperative strain endowed with capacitors of moderate volume prevails most of the time. Parameters: *N* = 40, *r* = 3.2, *g* = 5, *β* = 0.1, *μ* = 5 × 10^−5^, and *θ* = 0.12.

These explanations can be better understood in a quantitative way with the help of typical evolutionary paths, one of which is illustrated in Fig. 4. For conveniently describing this evolutionary path, we denote by *L*ow, *M*iddle, and *H*igh level when the average phenotypic diversity is located in the interval (0, 5], (5, 40], and (40, 50], respectively. Denote by *C*_*L*_ the state of cooperators with *L*ow average phenotypic diversity, and the like. With respect to this typical evolutionary path, five states emerge: *D*_*M*_, *C*_*M*_, *C*_*M*_ + *D*_*L*_, *C*_*M*_ + *D*_*M*_, and *C*_*M*_ + *D*_*H*_, and their corresponding fractions are respectively 0.659%, 99.099%, 0.0139%, 0.1899%, and 0.0389%. When the population is occupied by *C*_*M*_, the dynamics are extremely stable and stay put with a probability as high as 99.997%. It can but very rarely enter into either of the states *C*_*M*_ + *D*_*L*_, *C*_*M*_ + *D*_*M*_, *C*_*M*_ + *D*_*H*_ with probabilities 0.0002%, 0.0023%, and 0.0006%, respectively. Whenever residing in either of these three states, the population transits to the state *C*_*M*_ with the probabilities 1.4347%, 1.2113%, and 1.4918%, correspondingly. Switching between these four states constitutes the core component of the population dynamics. As the rate of flowing into the state *C*_*M*_ is trivial compared with that of flowing away from it, the population spends extremely high fraction of time in this state, and thus explains the macroscopic observations. We would like to point out that the population starting with the state *D*_*M*_ can be stabilized at it with a very high likelihood of 99.997%, at least in this typical path, but transitions to this state are so meagrely few that this state can produce no essential effect in the long run.

So far, our results have shown that cooperation coevolves with phenotypic diversity under a wide range of conditions and that there exists an optimal level of phenotypic diversity best promoting cooperation. It pays for cooperators to expand their capacitors (see Fig. 3a), thereby improving their opportunity of establishing cooperation via novel phenotypes, as these new phenotypes serve as secret handshakes that are difficult for defector to discover and chase after. Linearly increasing cost of expanding capacitor prevents capacitor’s volume rising to infinity. Tradeoff between these two forces leads to symbiosis of optimal phenotypic diversity and highest fraction of such cooperative strain. Our results also show that evolved high levels of phenotypic diversity can occasionally collapse due to the invasion of defector mutants, illustrating that cooperation and phenotypic diversity can mutually reinforce each other.

**Figure 5:**
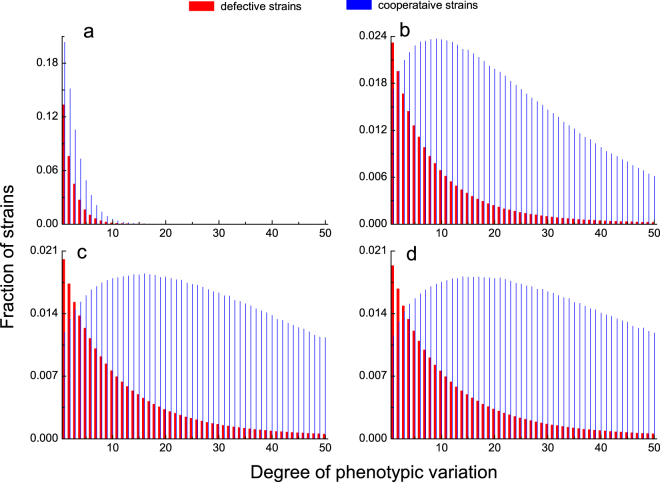
Stationary distribution for 2*M* competing strains for different time scales. The bars are obtained by solving the eigenvector of the 2*M* × 2*M* transition matrix. Blue denotes cooperative strain, and red defective strain. The abscissa value represents the volume of capacitor. The evolutionary process is fully characterized in the main text. In panel a, whenever interactions happen slowly, accumulated payoffs resulting from game interactions are low. High cost of carrying many phenotypes cannot be counteracted. Therefore, strains carrying too many phenotypes shall be easily eliminated. Evolutionary competition mainly progresses between strains carrying a small number of phenotypes. Even so, cooperative strains are likely to dominate the evolutionary race. In panels b, c and d, whenever rate of interaction is comparable, or even faster than that of selection, the evolutionary dynamics exhibit similar properties. Fraction of defective strain monotonically decreases as phenotypes this kind of strain carries increases. For cooperative strain, there exists an optimal level of phenotype to carry and corresponding cooperative strain accounts for the highest fraction. Parameters: *N* = 40, *r* = 3.2, *β* = 0.1, and *θ* = 0.12. In **a,b,c,** and **d,** *χ*(*x*) = 0.01*x*, 0.5*x*, 2*x*, 5*x*, correspondingly, and the overall cooperation levels are 0.675, 0.805, 0.784, and 0.787, respectively.

We have also considered the effects of different time scale of interaction to selection on the stationary distributions of strains. Figure 5 shows that our main results still hold true as long as public goods interactions happen fast enough. One can also find that whenever interactions happen slowly, effects of game interactions are weakened. Strains carrying many phenotypes cannot offset the cost of carrying so many phenotypes and shall be eliminated soon. Evolutionary competition mainly unfolds between strains carrying a small number of phenotypes. Even so, cooperative strains are likely to dominate the evolutionary race (see Fig. 5a). In contrast, whenever rate of interaction is comparable (see Fig. 5b), or even faster than that of selection (see Fig. 5c and d), the evolutionary dynamics exhibit similar properties. Fraction of defective strain monotonically decreases as phenotypes this kind of strain carries increases. For cooperative strain, there exists an optimal level of phenotype to carry and corresponding cooperative strain accounts for the highest fraction. Thus, our results provide new insights into better understanding the coevolution of cooperation and phenotypic diversity.

## Discussion

How to understand the rise and maintenance of altruistic behavior is a key problem in evolutionary biology^22,24–33^. Our study provides a possible path for cooperation to get established. Our study falls into the scope of chromodynamics of cooperation^5,6^, but is decisively different from previous studies concerning this topic. Generally speaking, the essence of chromodynamics is through decelerating the cooperator-defector interaction to promote cooperation. Ways achieving this purpose are various^3–6,10^, such as loosing the coupling of tag and strategy^5^ and prescribing high levels of phenotypic diversity^6^. The mechanism we proposed that inherently carrying diverse phenotypes while expressing one plays a similar role as theirs^5^. In this sense, our mechanism is paralleled to the ones proposed in refs^5,6^ and thus adds to the ‘other mechanisms that can accomplish the same stabilizing effect’ as the authors of ref.^5^ have suggested.

Furthermore, our model is verily like the one proposed in ref.^34^ in that interactions are local and competition is global. Group (or subpopulation) size changes in significantly different ways. In their model^34^, group size arriving at a threshold divides with certain probability and thus the group selection is involved. Our model does not involve group selection. Subpopulation expands or shrinks, entirely relying on the frequency-dependent selection. Though the significant difference, group splitting and inherent phenotypic variations both serve as avenues orienting the evolution of cooperation. Whether natural selection acts on the population just on the individual level as suggested in our model, or also by invoking group selection as assumed in the model of Traulsen *et al*., should be carefully treated.

Under natural selection, phenotypic diversity may be maintained by various mechanisms including mutant games^35^, sexual selection^36^, coevolving host-parasite population^37^, occasional recombinations of tags and of strategies^5^, and phenotype noise^11,15–19,38^. In our model, the population equilibrates at high levels of phenotypic diversity when driven by the frequency-dependent evolutionary dynamics. Our model does not prescribe any given phenotypic diversity but lets individuals carry a capacitor of potentially expressible phenotypes. Capacitor volume is subject to evolutionary force. Phenotypic diversity is therefore an evolvable trait. On this front, our results provide an adaptive explanation for the phenotypic diversity.

Our model marries two recurrent themes in evolutionary biology: the evolution of cooperation and inherent phenotypic variations. This combination may well capture the rationale underlying many observations in biological circles. For example, Salmonella Typhimurium can be either cooperative by expressing virulence, or non-cooperative by not expressing^20,21^. This expression does determine strategy. But most likely it also sends signal to others who have not expressed the virulence. In fact, by a mathematical model Ackermann *et al*. have pointed out that phenotype noise itself is insufficient to evolve cooperation^20^. The side-blotched male lizards exhibiting diverse colors in throats may be another pertinent example. Our model philosophy may be conceived in the process of evolving this property^39,40^. These conjectures have implications for and awaits the confirmation of field studies.

## Methods

### Model description

Consider a well mixed population of finite size (=*N*). These *N* individuals compete to survive by reproducing asexually. Each individual *i* is characterized by a triplet (*H*_*i*_, *S*_*i*_, *K*_*i*_), where *H*_*i*_ is the current phenotype expressed, *S*_*i*_ is the behavioral strategy, and *K*_*i*_ is the volume of individual *i*’s capacitor, which contains a number of phenotypes individual *i* can potentially express. Here *S*_*i*_ is the probability that *i* cooperates when the public goods game happens. We limit our attention to two strategies, cooperation (*S*_*i*_ = 1) and defection (*S*_*i*_ = 0). It is straightforward to extend strategy to the full space as done in ref.^41^.

Each individual randomly expresses one phenotype from her capacitor while the rest are phenotypically silent. Only phenotypes expressed are visible and thus the population is divided into a number of subpopulations. Each subpopulation consists of individuals expressing the same phenotype (see Fig. 1).

Individuals in the same subpopulation interact by playing the public goods games. In this sense, phenotypes play the role of assortment. We assume that the number of group interactions happening in each subpopulation is proportional to the size of this subpopluation. When a subpopulation size is less than the group size as required for the public goods game to happen, no interaction takes place. Individuals need to bear the cost of retaining capacitor and expressing phenotypes. The cost is assumed to be proportional to the volume of her capacitor, that is, *κ*_*i*_(*K*_*i*_) = *θK*_*i*_. Here we choose the simplest possible phenotype cost function. The net payoff *π*_*i*_ determines the reproductive success (fitness) *f*_*i*_ of an individual *i*. Here the fitness is an exponential function of payoff ${f}_{i}={e}^{\beta {\pi }_{i}}$, where *β* is the intensity of selection.

The evolutionary updating occurs according to a frequency-dependent Moran process. At each time step, an individual is chosen with probability proportional to its fitness to reproduce an offspring. Following birth, a randomly selected individual in the population dies. The population size is thus constant throughout the evolution. Reproduction is however subject to mutation. As a first attempt, we would like to address whether cooperation can emerge and persist when individuals can carry redundant expressible phenotypes. Therefore, we assume that mutation is completely random so that any two different kinds of strains are mutaully accessible by mutation, and thus effects of mutation mode are unbiased. When mutation happens (with probability *μ*), the offspring randomly adopts one of the two behavioral strategies and also acquires a capacitor of volume ${K^{\prime} }_{i}$ at a cost $\theta {K^{\prime} }_{i}$. Here ${K^{\prime} }_{i}$ is picked up from the set {1, 2, ..., *M*} equiprobably and *M* is the largest allowed capacitor. The mutant expresses one phenotype at random from this new capacitor, which we call random phenotype switching.

When it comes to the public goods game, *g* individuals decide whether or not to contribute to a common pool. A cooperator contributes *c* to the pool, while a defector contributes nothing. The sum contributed is multiplied by the factor *r* and then equally distributed to these *g* individuals, no matter whether they have contributed. Thus the payoff for a cooperator is $\frac{rjc}{g}-c$ and that for a defector is $\frac{rjc}{g}$ when there are *j* cooperators in the group. Without loss of generality and for simplicity^42^, we fixate the cost *c* of cooperation as 1. Under the classic constraint 1 < *r* < *g*, group of cooperators outperforms group of defectors whereas defectors are better off than cooperators in any given mixed group. Social dilemma arises as the best strategy for a player conflicts with the best strategy for the group.

We would like to point out that our present study can recreate qualitatively similar results presented in our previous work^38^ by setting the group size as *g* = 2. However, group interactions cannot be simply regarded as the sum of pairwise interactions. Group interaction cannot proceed in this way that all possible groups would play the public goods game^43,44^. Otherwise payoffs from games are likely to be unrealistically high. More importantly, occurrence of group interaction requires higher complexity^10,45–50^.

### Fixation probability

We here present the general procedure of calculating the fixation probability in brevity. For sufficiently small mutation rate, the population simultaneously admits at most of two different types of individuals, say *A* and *B*. Here ‘different’ means that at least an element of the triplet (*H*_*A*_, *S*_*A*_, *K*_*A*_) differs from that of the triplet (*H*_*B*_, *S*_*B*_, *K*_*B*_). We should bear in mind that *K*_*A*_ denotes the volume of *A*’s capacitor, and *S*_*A*_ her strategic behavior. *K*_*B*_ is *B*’s volume of capacitor and *S*_*B*_ her strategic behavior. *S*_*A*_ takes one if *A* is a cooperator, and zero otherwise. It is the same case with *S*_*B*_. Denote by *i* the number of *A* s in the population. The Moran process describing the evolutionary race has two absorbing states: *i* = 0 and *i* = *N*. When the population arrives at either of the two states, it will stay there forever. Denote by *ϕ*_*i*_ the fixation probability that the population eventually ends up at the state *i* = *N* when starting with the state *i*.

When *A* and *B* have expressed the same phenotype, they are located in the subpopulation (also the whole population) and would play the public goods game. At this time, we can easily get their expected payoffs respectively as $${P}_{A}=\chi (N)\cdot \{\frac{r}{g}[(1+\frac{(g-1)(i-1)}{N-1}){S}_{A}+(g-1-\frac{(g-1)(i-1)}{N-1}){S}_{B}]-{S}_{A}\}-\theta {K}_{A}$$ and $${P}_{B}=\chi (N)\cdot $$$$\{\frac{r}{g}[\frac{(g-1)i}{N-1}{S}_{A}+(g-\frac{(g-1)i}{N-1}){S}_{B}]-{S}_{B}\}-\theta {K}_{B}$$. When *A* and *B* have expressed different phenotypes, they are located in different subpopulations. Public goods interactions just happen in each of the two subpopulations. In this situation, the payoff for an *A* and a *B* can be respectively written as *P*_*A*_ = *χ*(*i*) · (*r* − 1)*S*_*A*_*I*_(*i*>=*g*)_ − *θK*_*A*_ and *P*_*B*_ = *χ*(*N* − *i*) · (*r* − 1) *S*_*B*_*I*_(*N*−*i*>=*g*)_ − *θK*_*B*_. Indication function *I*_(*i*>=*g*)_ means that only when the subpopulation size arrives at least the group size can interactions take place. Otherwise, no interaction happens. The function *χ*(*i*) describes how the number of interactions depends on the subpopulation size. We choose linear function *χ*(*x*) = 0.1*x*. We shall consider other time scales^51,52^ of interaction to selection later in this work. The fitness for *A* and *B* reads $${f}_{A}={e}^{\beta {P}_{A}}$$ and $${g}_{B}={e}^{\beta {P}_{B}}$$, respectively. The intensity of selection *β* measures how much payoff contributes to fitness. In an updating event, the population can add one, reduce one, remain the same in terms of the number of *A* s. The probability of increasing one, decreasing one is $${T}_{i,i+1}=\frac{i{f}_{A}}{i{f}_{A}+(N-i){g}_{B}}\,\cdot \,\frac{N-i}{N}$$, $${T}_{i,i-1}=\frac{(N-i){g}_{B}}{i{f}_{A}+(N-i){g}_{B}}\,\cdot \,\frac{i}{N}$$ and of remaining unchanged is *T*_*i*,*i*_ = 1 − *T*_*i*,*i*+1_ − *T*_*i*,*i*−1_. Then we have

$${\varphi }_{i}={T}_{i,i+1}{\varphi }_{i+1}+{T}_{i,i-1}{\varphi }_{i-1}+{T}_{i,i}{\varphi }_{i}$$

Under boundary conditions *ϕ*_0_ = 0 and *ϕ*_*N*_ = 1, we can derive the fixation probability as

$${\varphi }_{1}={(1+\sum _{l\mathrm{=1}}^{N-1}\prod _{k\mathrm{=1}}^{l}\frac{{T}_{k,k-1}}{{T}_{k,k+1}})}^{-1}$$

### Transition rate for pairwise competing strains

We here demonstrate the rate the population transits from *X* to *Y*, which means the probability that strain *X* as mutant invades and takes over the population of *Y* strain. We would like to point out that though individuals may express different phenotypes, as long as they carry the same number of potentially expressible phenotypes, they belong to the same kind of strain. Suppose strain *X*’s volume is *K*_*X*_ and strain *Y*’s volume is *K*_*Y*_. Denote by ${\rho }_{X\to Y}^{s}$ the fixation probability that a single mutant *X* takes over the resident population *Y* when *X* and *Y* happen to express the same phenotype, and by ${\rho }_{X\to Y}^{d}$ the fixation probability that a single mutant *X* takes over the resident population *Y* when *X* and *Y* have expressed different phenotypes.

Then the expected transition rate (omitting the mutation rate *μ* for notational brevity) from state *X* to *Y*, *r*(*X*, *Y*;*K*_*X*_, *K*_*Y*_), is given by

1 $$\begin{array}{rcl}r(X,Y;{K}_{X},{K}_{Y}) & = & H({K}_{Y}-{K}_{X})[\frac{1}{2}{\alpha }_{Y}{\rho }_{Y\to X}^{s}+\frac{1}{2}(1-{\alpha }_{Y}){\rho }_{Y\to X}^{d}]\\  &  & +[1-H({K}_{Y}-{K}_{X})]\{\frac{1}{2}\frac{{K}_{X}-{K}_{Y}}{{K}_{X}}{\rho }_{Y\to X}^{d}\\  &  & +\frac{1}{2}\frac{{K}_{Y}}{{K}_{X}}[{\alpha }_{Y}{\rho }_{Y\to X}^{s}+(1-{\alpha }_{Y}){\rho }_{Y\to X}^{d}]\}.\end{array}$$

*H* (·) is the Heaviside step function. *H*(*x*) = 1 if *x* ≥ 0, and *H*(*x*) = 0 if *x* < 0. Random phenotype switching means *α*_*X*_ = 1/*K*_*X*_, where *X*∈{*C*, *D*}. It is the same case with *α*_*Y*_. Some explanations can help understand the way of arriving at this transition rate. When the volume of *Y*’s capacitor exceeds that of *X*’s, strain *Y* with the likelihood *α*_*Y*_ expresses the same phenotype with strain *X*. The population moves from state *X* to state *Y* with the probability ${\rho }_{Y\to X}^{s}$. With probability 1 − *α*_*Y*_, strain *Y* expresses different phenotype from strain *X*. As this happens, the population moves from state *X* to state *Y* with the probability ${\rho }_{Y\to X}^{d}$. The coefficient $\frac{1}{2}$ means that the mutant can be equally likely a cooperator or a defector. Combining these possibilities together constitutes the first term in the right-hand side of *r*(*X*, *Y*; *K*_*X*_, *K*_*Y*_). When *K*_*Y*_ is less than *K*_*X*_, we can get the transition rate by considering two possibilities. If the phenotype that *X* has expressed is included in those *K*_*Y*_ phenotypes, the transition rate can be written down following the same logic as *K*_*Y*_ > *K*_*X*_. If the phenotype that *X* has expressed is not included in those *K*_*Y*_ phenotypes, *X* and *Y* are destined to be of different phenotypes, and the transition rate reads as $\frac{1}{2}\frac{{K}_{X}-{K}_{Y}}{{K}_{X}}{\rho }_{Y\to X}^{d}$. We can use Eq. (1) to analytically derive the transition rates between different population states in the limit of rare mutations and for any intensity of selection *β*.

### Stationary distribution

Individuals possessing capacitors of too large volume will be easily invaded by those who are endowed with capacitors of modest volume as cost of expanding volume increases linearly. In the long run, their fractions are nearly negligible. It thus makes sense to assume that the volume of capacitor is bounded by a finite number *M*. Combining with strategies, cooperation and defection, there are 2*M* different kinds of strains. In the limit in which mutations are rare^53^, the population will end up with the mutant either wiping out the residents or being wiped out by the residents. In other words, there are simultaneously at most two differing strains present in the population before next mutation occurs. Therefore, the population dynamics of 2*M* strains can be well approximated by an embedded Markov chain between these *M* full defective states and *M* full cooperative states. For convenience’s sake, we label cooperative strains with even numbers 2*K*_*C*_, and defective strains with odd numbers, 2*K*_*D*_ − 1, for 1 ≤ *K*_*C*_ ≤ *M* and 1 ≤ *K*_*D*_ ≤ *M*. For strain *X* possessing a capacitor of volume *K*_*X*_ and strain *Y* possessing a capacitor of volume *K*_*Y*_, the expected transition rate from state *X* to state *Y* is *r*(*X*, *Y*; *K*_*X*_, *K*_*Y*_) as shown by Equation (1) in Methods Section. We can then easily get the transition matrix *A* with dimension 2*M* by 2*M*. The *ij*^*th*^ entry of matrix *A* is *r*(*i*, *j*; *K*_*i*_, *K*_*j*_) for *i* ≠ *j*, and the *ii*^*th*^ entry is one minus the sum of all other entries in the *i* th row. It is worth noting that we have analytically derived the transition rates between any two competing strains and thus the transition matrix. The normalized left eigenvector associated with the eigenvalue 1 of the transition matrix *A* provides the stationary distribution of these 2*M* full states. The overall cooperative level can be obtained by summing all the elements with even indices in normalized eigenvector^54,55^. For slightly larger mutation rates, we can arrive at the population dynamics by the agent-based simulation.
